# Chemokine ligand 14 correlates with immune cell infiltration in the gastric cancer microenvironment in predicting unfavorable prognosis

**DOI:** 10.3389/fphar.2024.1397656

**Published:** 2024-06-03

**Authors:** Bing Lu, Jiawen Shi, Tong Cheng, Congshuo Wang, Manyu Xu, Pingping Sun, Xiaojing Zhang, Lei Yang, Peng Li, Han Wu, Xiaoling Kuai

**Affiliations:** ^1^ Department of Clinical Biobank, Nantong University Affiliated Hospital, Nantong, China; ^2^ Department of Pathology and Pathophysiology, School of Medicine, Nantong University, Nantong, China; ^3^ Department of Oncology, School of Medicine, Nantong University, Nantong, Jiangsu, China; ^4^ Department of Gastroenterology, Nantong University Affiliated Hospital, Nantong, Jiangsu, China; ^5^ Department of General Surgery, Nantong University Affiliated Hospital, Nantong, Jiangsu, China

**Keywords:** CCL14, tumor microenvironment, tumor-infiltrating immune cells, immune checkpoint, immunotherapy, chemokine drug target

## Abstract

**Objective:**

Gastric cancer (GC) is the world’s third-leading cause of cancer-related mortality; the prognosis for GC patients remains poor in terms of a lack of reliable biomarkers for early diagnosis and immune therapy response prediction. Here, we aim to discover the connection between chemokine ligand 14 (CCL14) expression in the gastric tumor microenvironment (TME) and its clinical significance and investigate its correlation with immune cell infiltration.

**Methods:**

We assessed CCL14 mRNA expression and its interrelation with tumor-infiltrating immune cells (TILs) using bioinformatics analysis in gastric cancer. CCL14 protein expression, TILs, and immune checkpoints were detected by multiple immunohistochemistry analyses in gastric cancer tissue microarrays. Then, we conducted statistics analysis to determine the association between CCL14-related patient survival and immune cell infiltration (*p* < 0.05).

**Results:**

We found that the CCL14 protein was separately expressed in the carcinoma cells and TILs in stomach cancer tissues. The CCL14 protein was related to tumor differentiation and tumor depth and positively correlated with the presentation of LAG3 and PD-L1 in gastric cancer cells. In addition, the CCL14 protein in the TILs of gastric cancer tissues was related to Lauren’s type cells, T cells (CD4^+^ and CD8^+^), and CD68^+^ macrophages in the TME. Kaplan–Meier survival and multivariate analyses showed that the CCL14 expression in gastric cancer cells was an independent prognostic factor.

**Conclusion:**

Our study illustrated that CCL14 is a poor prognosis biomarker in gastric cancer, which may be associated with the potential for immunotherapy.

## 1 Introduction

Gastric cancer (GC) is ranked as the third most prevalent cause of cancer-related death globally and the fifth most diagnosed cancer worldwide. The rapidly increasing incidence and mortality of gastric cancer is an economic burden and a threat to public health (Kelly et al., 2021). Immunity is important in the defense against infectious agents and tumor monitoring and is critical in tumor genesis and development (Sethi et al., 2019). The survival outcome of advanced gastric cancer patients is poor even after operation and chemotherapy. Recently, immunotherapy shed light on anti-tumor cells. It is not always effective because of the complicated tumor heterogeneity and tumor microenvironment (TME) (Wang et al., 2021). A better understanding of the TME of gastric cancer will improve the clinical practice of immunity therapy.

The TME comprises neoplastic cells, tumor-infiltrating immune cells (TILs), stromal cells, and vascular endothelial cells. Recent advanced studies demonstrated that the TME is critical in tumor progression (Wu et al., 2018; Wang and Zhang, 2021). Immune cell recruitment into the TME is mediated by chemokines (Nagarsheth et al., 2017). Thus, chemokines are essential in the formation of the TME. Furthermore, most chemokines also promote tumor growth and metastasis (Hanahan and Weinberg, 2011; Kalbasi et al., 2017), while some chemokines have opposite functions (Lesina et al., 2016; Zhu et al., 2019).

CC chemokine ligand 14 (CCL14), which occurs in many tissues, is a CC-type chemokine that was first identified in chronic renal failure patients (Schulz-Knappe et al., 1996). CCL14 consists of 74 amino acids and has a molecular weight of 8,673 KDa (Blain et al., 2007). Interestingly, 46% of the CCL14 sequence is homologous to the macrophage inflammatory protein (Schulz-Knappe et al., 1996). CCL14 induces the chemo-axis of immune cells by binding to its receptors (Nagarsheth et al., 2017). Although CCL14 was reported to play a role in breast cancer and hepatocellular carcinoma progression as a prognostic biomarker (Li et al., 2011; Gu et al., 2020), CCL14 expression in the TME of gastric cancer is not fully known.

In the current study, we performed CCL14 representation and studied its interrelation with gastric cancer prognosis. Moreover, we investigated the relevance of CCL14 expression to different types of immune cells and immune checkpoints. Our results demonstrated that CCL14 is a prospective therapeutic for gastric cancer.

## 2 Materials and methods

### 2.1 Expression and prognostic analysis of CCL14 mRNA in gastric cancer

We obtained 300 tumor samples and 100 normal samples from the GEO database (GSE62254 and GSE66222) (https://www.ncbi.nlm.nih.gov/gds). In addition, GEPIA (http://gepia.cancer-pku.cn/index.html) was taken to examine the interrelation between CCL14 mRNA expression and gastric cancer survival. The CCL14 mRNA expression with matched clinical information of 375 gastric tumor tissues and 32 benign gastric mucosa epithelial tissues was acquired from the publicly available database TCGA. GEPIA is a web service used to analyze RNA sequencing representation data generated by TCGA and GTEx programs (Li et al., 2021).

The differential representation of CCL14 mRNA between gastric cancer and non-tumorous tissues was graphed with boxplots. The Kaplan–Meier curve was applied to identify the relation between CCL14 mRNA and gastric cancer prognosis.

### 2.2 Correlation analysis of CCL14 mRNA and immune cells

The TIMER database contains a systematic analysis of TILs for 32 cancer categories in over 10,000 samples from TCGA (https://cistrome.shinyapps.io/timer/). The TIMER database detected the abundance of TILs based on the statistical analysis of gene representative signatures (Li et al., 2017). We evaluated the relevance between CCL14 expression and six categories of TILs, including B cells, T cells (CD4^+^ and CD8^+^), neutrophils, dendritic cells, and macrophages.

### 2.3 Clinical samples

The tissue microarray (TMA), including 133 gastric cancer and 67 benign gastric mucosa tissues, was obtained from Clinical Biobank, Affiliated Hospital of Nantong University. The tissues were gathered from patients who underwent operations from June 2004 to July 2009. All the patients did not undergo chemotherapy, radiation therapy, or immunotherapy before the operation in this study. The pathological diagnosis of gastric cancer was evaluated based on Lauren’s type classification of the 5th World Health Organization standards. This study obtained approval from the Ethics Committee of Nantong University Hospital.

### 2.4 Immunohistochemistry and multiplex fluorescence IHC

The 4-μm slides were cut from the gastric TMA. At room temperature, the slices were de-waxed using xylene and rehydrated in gradient alcohol and distilled water. Then, they were fixed at room temperature for 10 min with 10% formalin. Then, the sections were microwave-cooked in AR6 buffer (AR600250ML, Akoya Biosciences, United States) to repair antigens, and then, they were placed in an antibody-blocking solution (ARD1001EA, Akoya Biosciences, United States) for 10 min to block unspecific binding. The slides of the TMA were incubated with a primary antibody overnight at 4°C, washed three times with TBST, and then incubated for 10 min with a secondary antibody. The sections were stained as previously described (Jiang et al., 2021; Zhang et al., 2022). For multiplex fluorescence IHC (mIHC), the staining was repetitively inactivated by microwave treatment. After the final antibody staining, the slides were washed and mounted with 6-diamidino-2-phenylindole (DAPI; F6057, Sigma-Aldrich, United States).

The following major antibodies were utilized: anti-CCL14 antibody (1:50; sc-376152, santa, United States), anti-CD3 antibody (1:200; 85061, CST, United States), anti-CD4 antibody (1:200; ab133616, Abcam, United Kingdom), anti-CD8 antibody (ab93278, Abcam), anti-CD66b (1:500; arg66287, Airgobio, China), anti-CD68 antibody (1:400;76437, CST, United States), anti-CTLA4 antibody (1:50; NB100-64849, Novus, United States), anti-LAG3 antibody (1:50; 15372, CST, United States), anti-PD-1 antibody (1:50; 86163, CST, United States), anti-PD-L1 antibody (1:50; CST, United States), and anti-cytokeratin antibody (1:400; orb69073, Biorbyt, United Kingdom). All these slides were swept using an automated quantitative imaging system (Vectra 3.0, PerkinElmer/Akoya, United States) to determine the expressions of biomarkers. The images were analyzed using quantification software (inForm 2.4.1, PerkinElmer/Akoya, United States). The machine software application was trained to identify the areas of tumor and stromal tissue and recognize and quantify the cell types by specific labeling.

The immunohistochemistry (IHC) (CCL11, CTLA4, and PD-L1) staining strength was scored as 0, 1, 2, and 3, representing no staining, weak staining, moderate staining, and strong staining, respectively. The positive rate ranged from 0 to 100. The ultimate IHC score was computed as the total sum of the staining strength and positivity rate. Thus, the staining score was between 0 and 300. The significant cutoff point of CCL14 protein expression was identified using X-tile software (Rimm Lab in Yale University, United States) via the survival time of the patients. The staining score of TILs or immune checkpoints was the proportion of certain positive fluorescent cells/cancer cells in the tumor areas. The expression score was the ratio of the positive cells to all cells of the scanned field.

### 2.5 Statistical analysis

We analyzed the interrelation between CCL14 representation and clinical characteristics with Student’s *t*-test and χ^2^ tests for clinical tissue samples. Univariate and multivariate analyses were used for Cox regression to evaluate the prognostic factors. Kaplan–Meier analysis was adopted to perform the analysis of survival. Pearson’s correlation was performed for relation analysis. We adopted these statistical analyses using the SPSS 20.0 software package (SPSS, Chicago, United States). *p-*values less than 0.05 were regarded as statistically meaningful. The R-value of the correlation statistics was considered a clear correlation for values more than 0.4.

## 3 Results

### 3.1 The significance of the CCL14 mRNA expression and outcome in gastric cancer

**FIGURE 1:**
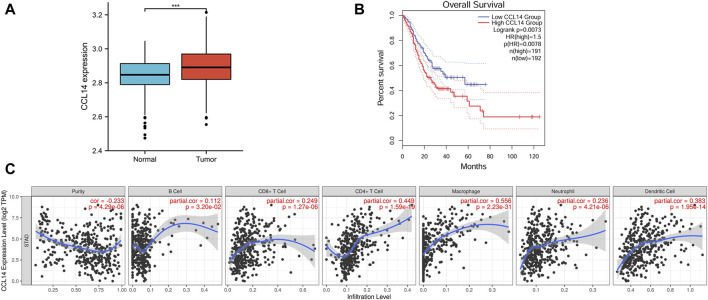
Bioinformatics analysis results of CCL14 mRNA in gastric cancer (GC). **(A)** Expression of CCL14 in gastric cancer tissues was lower than in benign gastric tissues. **(B)** Kaplan–Meier analysis with the GEPIA database showed high expression of CCL14 mRNA (red line) and low expression of CCL14 mRNA (blue line). **(C)** CCL14 mRNA expression was related by varying degrees to B cells, CD8^+^ T cells, CD4^+^ T cells, macrophages, neutrophils, and dendritic cells.

CCL14 mRNA expression was significantly higher in gastric cancer tissue compared to benign stomach tissue using data downloaded from the GEO database, which included 300 gastric cancer samples and 100 non-malignant samples (Figure 1A).

We also characterized the prognostic value of CCL14 mRNA expression for gastric cancer. The conclusions demonstrated that the higher expression of CCL14 in gastric cancer cells was associated with a poor prognosis (*p* = 0.008; Figure 1B).

CCL14 was one of the chemokines that induced the chemo-axis of immune cells. Therefore, we demonstrated the relationship of mRNA expression between CCL14 and TILs in gastric cancer tissues. The results of the analysis using the TIMER database indicate that CCL14 expression is associated with B cells (*r* = 0.112, *p* = 3.20e-02), CD8^+^ T cells (*r* = 0.249, *p* = 1.27e-06), CD4^+^ T cells (*r* = 0.449, *p* = 1.59e-19), macrophages (*r* = 0.556, *p* = 2.23e-31), neutrophils (*r* = 0.236, *p* = 4.21e-06), and dendritic cells (*r* = 0.383, *p* = 1.95e-14) (Figure 1C).

### 3.2 CCL14 protein localization and expression in gastric cancer tissues

**FIGURE 2:**
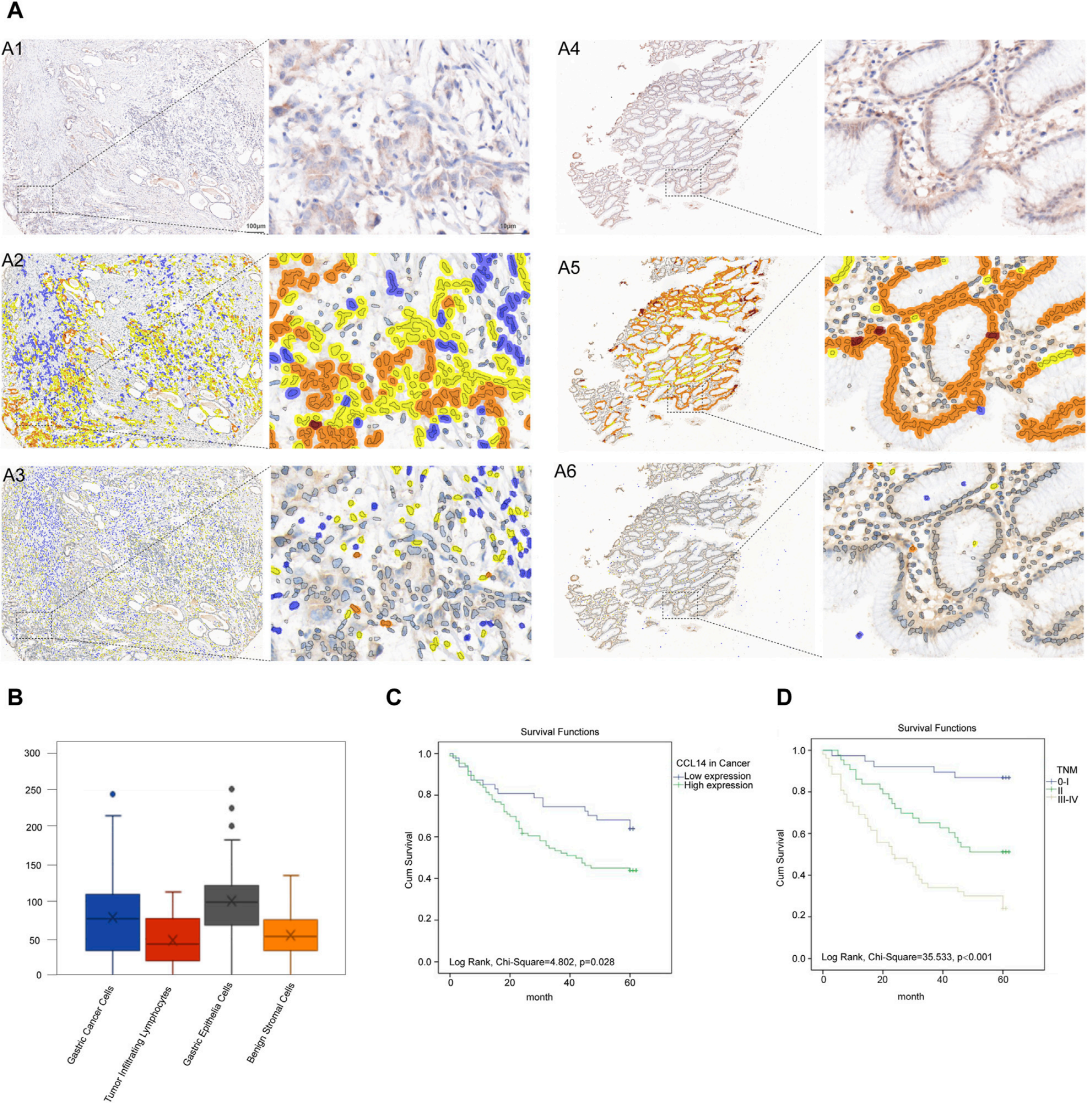
CCL14 protein expression in gastric cancer tissues. **(A)** Representative patterns of CCL14 immunoreactivity in gastric cancer and gastric epithelial tissues were scored using software (intensity: brown, intense staining; orange, moderate staining; yellow, weak staining; and blue, no staining). A1: CCL14 expression in gastric cancer tissues. A2: CCL14 expression in gastric cancer cells. A3: CCL14 expression in tumor-infiltrating immune cells (TILs) of gastric cancer. A4: CCL14 expression in gastric mucosa tissues. A5: CCL14 expression in gastric epithelial cells. A6: CCL14 expression in immune cells of the gastric mucosa (left column magnification ×20, and right column magnification ×400). **(B)** Score (0–300) of CCL14 expression in gastric cancer cells, TILs, gastric epithelial cells, and benign gastric stromal cells. **(C,D)** Survival curves of gastric cancer patients by Kaplan–Meier analysis. **(C)** High CCL14 protein expression (green line), and low CCL14 protein expression (blue line). **(D)** TNM 0–I stages (blue line), TNM II stage (green line), and TNM III–IV stages (yellow line).

Since mRNA expression does not predict protein levels, there is no linear relation between the two in biological samples (Pascal et al., 2008; Maier et al., 2009; Liu et al., 2016). We used immunohistochemical analysis to investigate the expression of the CCL14 protein and other immune-related markers in TMA sections containing 133 gastric cancer tissues and 67 benign gastric mucosa tissues. CCL14-positive staining was preponderantly localized in gastric cancer cells, gastric mucosal epithelial cells, TILs, and immune cells in the gastric mucosa, which was found using imaging software (Figure 2A).

We ascertained statistically significant lower expression of the CCL14 protein in gastric cancer cells than in gastric mucosal epithelial cells (79.43 ± 54.80 vs. 100.75 ± 48.17, *p* = 0.008) (Figure 2B). The result was coincident with the results of the GEPIA analysis. We demonstrated that CCL14 protein expression was lower in TILs than in benign gastric stromal cells (48.58 ± 31.983 vs. 55.55 ± 28.55), with an insignificant difference (*p* = 0.137) (Figure 2B). Furthermore, the statistics results indicated no relation between CCL14 expression in cancer cells and TILs in gastric cancer tissues (*r* = 0.569, *p* = 0.133) via Pearson’s correlation.

### 3.3 The association between CCL14 expression and pathological features in gastric cancer

Fortunately, the cutoff point of CCL14 protein expression in stomach cancer cells or TILs was 50 according to the outcome of these gastric cancer patients using X-tile software. Scores of 0–50 and 51–300 indicated low and high expression, respectively. The relation between the CCL14 protein and clinical features of the patients of gastric cancer is shown in Table 1. It is clear that CCL14 protein expression in gastric cancer cells is interrelated to differentiation (*p* = 0.026) and tumor depth (*p* = 0.034). The CCL14 protein expression in TILs was associated with Lauren’s classification (*p* = 0.005) (Table 1).

**TABLE 1:** Correlation between the expression of CCL14 and tumor-infiltrating immune cells (TILs) and the pathological characteristics of patients with gastric cancer.

Characteristic	n	CCL14 in cancer cells		CCL14 in TILs			
		High n (%)	Pearson’s χ^2^	*p*	High n (%)	Pearson’s χ^2^	*p*
Total	133	86 (64.66)			60 (45.11)		
Gender			1.966	0.161		0.580	0.446
Male	100	68 (68.00)			47 (47.00)		
Female	33	18 (54.55)			13 (39.39)		
Age			0.222	0.638		0.462	0.497
<60	53	33 (62.26)			22 (41.51)		
≥60	80	50 (66.25)			38 (47.50)		
Lauren’s type			0.185	1.755		7.816	0.005*
Intestinal type	115	72 (62.11)			48 (41.47)		
Diffuse type + mixed	15	12 (80.00)			12 (80.00)		
Other^a^	3	2			0		
Differentiation			4.983	0.026*		0.412	0.521
Well and moderate	44	33 (75.00)			19 (43.18)		
Poor	70	38 (54.29)			26 (37.14)		
Other^b^	19						
HER-2			3.023	0.388		1.491	0.684
Negative	101	62 (61.39)			43 (42.57)		
Poor positive	11	7 (63.64)			6 (54.55)		
Moderate positive	14	11 (78.57)			8 (57.14)		
Strong positive	7	6 (85.71)			3 (42.86)		
Preoperative CA199			0.008	0.931		0.843	0.359
≤37 U/mL	64	41 (64.06)			29 (45.31)		
>37 U/mL	8	5 (62.50)			5 (62.50)		
Unknown	61	40			26		
T			6.746	0.034*		0.419	0.811
Tis-T1	23	18 (78.26)			10 (43.48)		
T2	25	11 (44.00)			10 (40.00)		
T3–4	85	57 (67.06)			40 (47.06)		
N			1.836	0.399		3.730	0.155
N0	61	36 (59.02)			22 (36.07)		
N1	23	15 (65.22)			12 (52.17)		
N2–3	49	35 (71.43)			26 (53.06)		
M			0.011	0.916		1.179	0.278
M0	127	82 (64.57)			56 (44.09)		
M1	6	4 (66.67)			4 (66.67)		
TNM stage			0.844	0.656		1.005	0.605
0–I	38	24 (63.16)			10 (39.47)		
II	43	26 (60.47)			19 (44.19)		
III–IV	52	36 (69.23)			26 (50.00)		

*p < 0.05, and bold values indicated significance.

Others^a^: adeno-squamous carcinoma, two cases and squamous cell carcinoma, one case.

Others^b^: besides intestinal type adenocarcinoma.

Multivariate and univariate analyses were performed to detect the relative factors of the clinical outcome in gastric cancer patients. The univariate analysis demonstrated that the CCL14 representation in cancer cells, CCL14 representation in TILs, serum level CA199, tumor depth, lymph nodes, distant metastasis, and TNM stages were correlated with overall survival (Table 2). In multivariate analysis, the expression of CCL14 in cancer cells, preoperative CA199 level, and TNM stage were individual predictive factors (Table 2). The outcome of gastric cancer patients with high CCL14 expression in gastric cancer cells, high preoperative CA199 levels, and an advanced TNM stage had significantly dismal survival (Figures 2C and D).

**TABLE 2:** Correlation between CCL14 expression, clinicopathological characteristics, and patient overall survival.

Clinicopathological characteristics	Univariate analysis				Multivariate analysis			
	HR	*p* >|z|	95% CI		HR	*p* >|z|	95% CI	
CCL14 in cancer cells								
High vs. low	1.836	0.032*	1.054	3.197	5.823	0.007*	1.605	21.130
CCL14 in stromal cells								
High vs. low	1.668	0.040*	1.023	2.719	0.398	0.103	0.131	1.204
Age (years)								
≤60 vs. > 60	1.253	0.386	0.753	2.083				
Gender								
Male vs. female	0.869	0.618	0.499	1.511				
Lauren’s type								
Intestinal vs. diffuse + mixed	1.193	0.640	0.569	2.504				
Differentiation								
Well and middle vs. poor	0.916	0.749	0.533	1.572	1.432	0.439	0.577	3.557
HER-2								
Strong vs. middle vs. weak vs. negative	0.949	0.717	0.715	1.260				
Preoperative CA199								
≤37 vs. > 37	4.426	0.001*	1.907	10.274	7.230	0.002*	2.111	24.754
T								
Tis–1 vs. 2 vs. 3 vs. 4	2.298	<0.001*	1.483	3.561				
N								
N0 vs. N1 vs. N2 vs. N3	2.719	<0.001*	1.635	2.904				
M								
M0 vs. M1	8.847	<0.001*	3.679	21.274				
TNM stage								
0–I vs. II vs. III–IV	2.739	<0.001*	1.909	3.928	3.993	<0.001*	2.166	7.359

*p < 0.05.

### 3.4 Relevance of CCL14 expression to TILs or immune checkpoints

Chemokines in the immune system can mediate the infiltration of immune cells. In this study, CCL14 mRNA expression was linked to TILs in the TIMER dataset. The CD4^+^ T cells and CD68^+^ macrophages are positively related to some kinds of immune cells.

**FIGURE 3:**
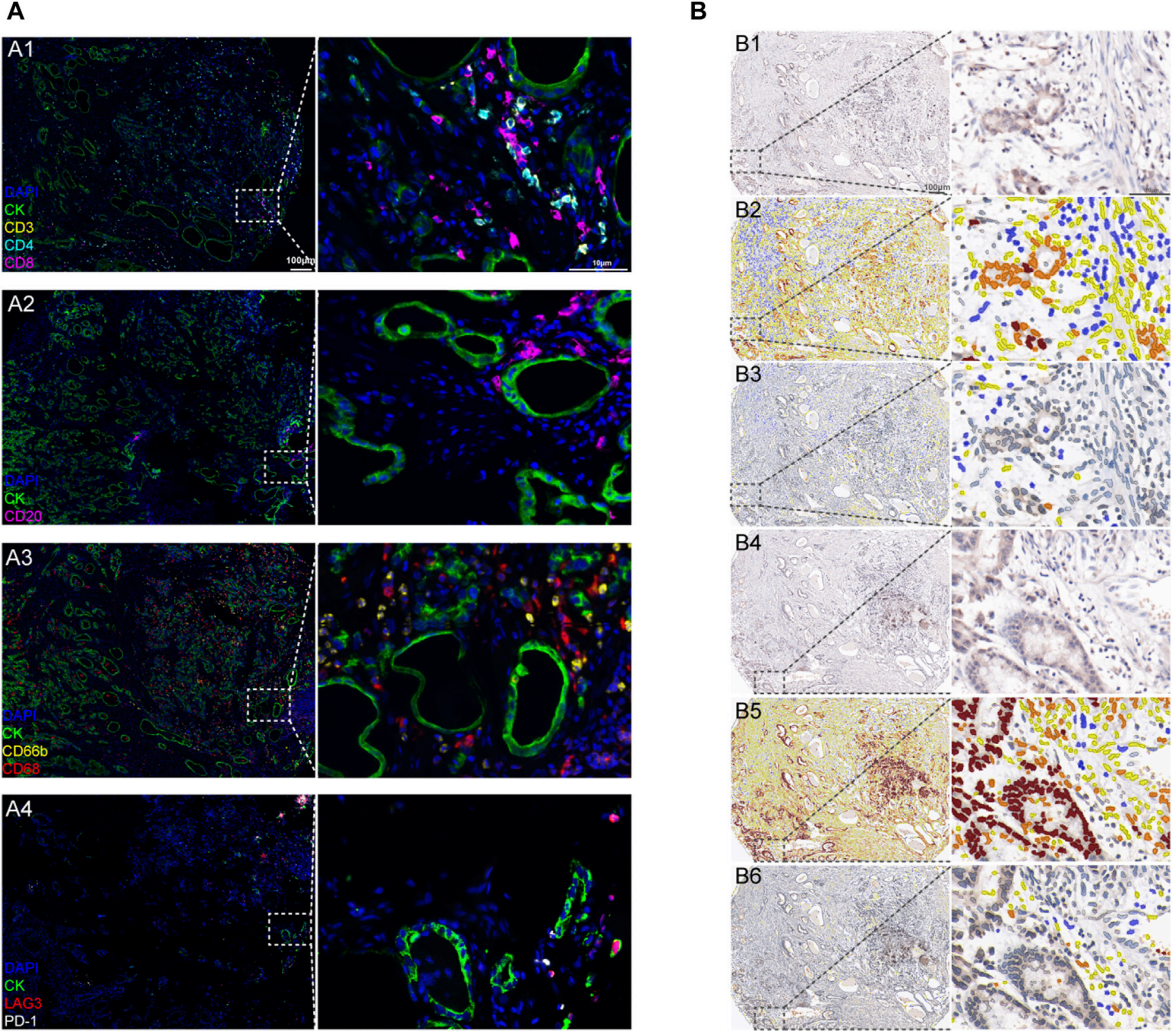
Representative immunoreactivity patterns of TILs and immune checkpoint expression in gastric cancer tissues: **(A)** GC immune environment using multiplex fluorescence IHC (mIHC) panels scanned using Vectra software. A1: lymphocyte panel of CD3, CD4, and CD8 with CK staining. A2: B lymphocyte panel of CD20 with CK staining. A3: myeloid panel of CD66B and CD68 with CK staining. A4: immune therapy target panel of PD-1 and LAG3 with CK staining (green: CK; blue: DAPI; left column magnification ×20; right column magnification ×400). **(B)** Quantified protein expression using the immunohistochemistry (IHC) score (intensity: brown, intense staining; orange, moderate staining; yellow, weak staining; and blue, no staining. Left column magnification ×20; right column magnification ×400). B1: CTLA4 in cancer tissues. B2: CTLA4 expression in cancer cells was scored using software. B3: CTLA4 expression in TILs was scored using software. B4: PD-L1 in cancer tissues. B5: PD-L1 expression in cancer cells was scored using software. B6: PD-L1 expression in TILs was scored using software.

We further accessed the expression of B cells, CD4^+^ T cells, CD8^+^ T cells, macrophages, neutrophils, and immune checkpoints (CTLA4, LAG3, PD-1, and PD-L1) in gastric cancer tissues using IHC and mIHC staining and multispectral image analysis. We used Pearson’s correlation to calculate the protein staining score of CCL14 expression and other markers mentioned above. CCL14 protein expression in cancer cells was positively related to LAG3 expression (*r* = 0.536, *p* < 0.001) and PD-L1 (*r* = 0.518, *p* < 0.001) (Figure 3; Table 3). At the same time, the CCL14 expression in TILs had a linear correlation with immune cells such as CD4^+^ T cells (*r* = 0.425, *p* < 0.001), CD8^+^ T cells (*r* = 0.406, *p* < 0.001), and CD68^+^ macrophages (*r* = 0.421, *p* < 0.001) (Figure 3; Table 3).

**TABLE 3:** Correlation between CCL14 expression and tumor-infiltrating immune cells (TILs) and immune checkpoints.

Gene markers	n	CCL14 in cancer cells		CCL14 in TILs	
		Pearson’s correlation	*p*	Pearson’s correlation	*p*
CD4^+^ T	121	−0.042	0.644	**0.425**	<0.001*
CD8^+^ T	117	0.137	0.140	**0.406**	<0.001*
CD20^+^	120	0.095	0.302	0.189	0.039*
CD66b^+^	124	0.105	0.244	0.269	0.003*
CD68^+^	124	0.286	0.001*	**0.421**	<0.001*
CTLA4^+^	125	0.080	0.373	−0.040	0.658
LAG3^+^	119	**0.536**	<0.001*	0.274	0.003*
PD-1^+^	119	0.208	0.023*	0.294	0.001*
PD-L1^+^	120	**0.518**	<0.001*	0.376	<0.001*

*p < 0.05, and bold values indicated significance.

## 4 Discussion

In recent years, even though the prognosis of early gastric disease has improved, patients with advanced gastric cancer still have a short overall survival time. The immune system is an imperative factor in the development, growth, and progression of cancer (Hanahan and Weinberg, 2011). Immunotherapy is promising. The host immune system is involved in the efficacy of immunotherapy (Topalian et al., 2012). Some patients benefit from immunotherapy, and some are not affected due to the complicated TME. In the TME, cytokines and chemokines regulate the infiltration of TILs. TILs are indispensable components of the TME and could predict the outcome of cancer treatment (Liu et al., 2017; Zhang S. et al., 2019; Zhang S. C. et al., 2019). Previous studies have also demonstrated that TILs are correlated with tumor genesis, invasion, and metastasis. This study investigated the expression of chemokine CCL14 and its relationship with TILs in gastric cancer cells through the bioinformatics analysis method and high-dimensional IHC staining with multispectral image analysis. Some types of TILs in the TME of gastric cancer were correlated with CCL14 expression. It is possible that CCL14 regulates the migration of TILs into gastric cancer tissues and affects the clinical outcome.

Chemokines are a group of small cytokines with chemotactic function that interact with transmembrane receptors and play a crucial role in many physiological processes, notably, recruiting cells to specific sites. CCL14 is a chemokine and has gained attention recently because it has the CCR5 receptor, which is a potent inhibitor of HIV (Detheux et al., 2000; Munch et al., 2002). Human tissues such as the liver, spleen, and myocardium have CCL14 and are involved in biological activities (Schulz-Knappe et al., 1996). CCL14 expression is related to the pathogenesis and progression of inflammation and cancer (Gupta et al., 2008; Zhu et al., 2019). High CCL14 expression is linked to long-term outcome in epithelial ovarian cancer patients (Cai et al., 2020) and inferior prognosis in hepatocellular carcinoma (Zhu et al., 2019).

This investigation explored the CCL14 expression in stomach cancer and its relation with the clinical outcome. Both the GEO database and IHC in the TMA showed that CCL14 expression was significantly elevated in gastric cancer cells and was associated with an inferior prognosis. Our results are not the same as that of other studies of CCL14 expression in hepatocellular carcinoma and epithelial ovarian cancer (Zhu et al., 2019; Cai et al., 2020). Because the role of the same gene or protein is not the same in different cancer types (Sack et al., 2018), we first investigated CCL14 protein expression in the gastric cancer TME and found that CCL14 is an outcome factor in gastric cancer. The survival plots also indicated that gastric cancer patients with a high expression of CCL14 in gastric cancer cells had a significantly shorter survival time. TILs are essential elements of the TME and are implicated in tumorigenesis, progression, invasion, and metastasis (Man et al., 2013). However, the clinical significance of the TILs in gastric cancer is still under exploration. This investigation also indicated that CCL14 expression in gastric cancer cells was relevant to differentiation types and the tumor status. In cancer, the role of chemokines is complex and involves leukocyte migration, stromal cells, and tumor cells. Different chemokine receptors are expressed in different TILs. In the TME, chemokines mediate TIL recruitment (Moser and Loetscher, 2001). Chemokines CXCL1 and CXCL5 in macrophages are critical in gastric cancer metastasis (Zhou et al., 2019). CXC chemokines are related to the recurrence and prognosis of gastric cancer and are also correlated with attracting tumor cells and anti-tumor cells (Chen et al., 2020). In our research, the statistical results of IHC demonstrated that CCL14 expression in TILs was predominantly positively relevant to CD4^+^ T cells, CD8^+^ T cells, and CD68^+^ macrophages, which is in agreement with the TIMER database. In tumor immunity, CD4^+^ T cells can activate CD8^+^ T cells through various mechanisms to differentiate into cytotoxic T lymphocytes (Miggelbrink et al., 2021). Other studies clarify that tumor-derived growth factors VEGF and endothelin-1 inhibit the expression of adhesion molecules and immobilize T cells into tumors (Motz et al., 2014).

The results of IHC revealed that CCL14 expression in cancer cells was related to immune checkpoints LAG3 and PD-L1. Some studies demonstrated that chemokines regulated TILs that were related to the reaction of immunotherapy, including anti-PD-1 therapy (Chow et al., 2019; Biasci et al., 2020; Bockorny et al., 2020). CXCL9 of tumor-associated macrophages inhibited CD8^+^ T-cell infiltration and impaired anti-PD1 treatment (Pascual-Garcia et al., 2019). The sustained activation of PD-1 induces T-cell exhaustion. Pd-1 binds to PD-L1 to regulate T-cell activation, which induces CD3 dephosphorylation and leads to T-cell exhaustion. CCL4, CCL5, CXCL9, and CXCL10 are associated with CD8^+^ T cells in pancreatic cancer (Romero et al., 2020). The efficacy of anti-PD1 therapy is related to CD8^+^ T cells. Chemokines can regulate CD8^+^ cell migration, so it associates with the effects of anti-PD1 treatment. Our research found that CCL14 was correlated with certain classes of immune cells and immune checkpoints, which could be a potential target for immune therapy.

There were some limitations to our research. First, we analyzed CCL14 expression in the TME of gastric cancer tissue, and our results lack cross-talk clues between the tumor cells and TILs. Second, the relationship between cancer-associated CCL14 expression and the TILs remains to be determined in order to find evidence that CCL14 and immune cells jointly exert anti-tumor function in gastric cancer cells. Finally, it is unclear whether CCL14 would also induce immune escape in *in vivo* gastric cancer models.

In this study, using the bioinformatics system and TMA-mIHC, we proved that CCL14 is a prospective biomarker of gastric cancer. In future, we need to clarify the mechanism of CCL14 in regulating TILs and immune checkpoints.

## Data Availability

Raw data were generated at the Clinical Bio-bank of the Affiliated Hospital of Nantong University. Derived data supporting the findings of this study are available from the corresponding author JL Shi upon request for the protection of patient privacy.
